# Effect of Cu supplementation on genomic instability in chemically-induced mammary carcinogenesis in the rat

**DOI:** 10.1186/1423-0127-18-95

**Published:** 2011-12-22

**Authors:** Barbara Bobrowska, Dorota Skrajnowska, Andrzej Tokarz

**Affiliations:** 1 Department of Bromatology, Medical University of Warsaw, Poland, Banacha 1, 02-097 Warsaw

## Abstract

**Backround:**

The aim of the present study was to assess the effect of dietary supplementation (copper or copper and resveratrol) on the intensity of carcinogenesis and the frequency of microsatellite instability in a widely used model of mammary carcinogenesis induced in the rat by treatment with 7,12-dimethylbenz[a]anthracene (DMBA).

**Methods:**

DNA was extracted from rat mammary cancers and normal tisues, amplified by PCR, using different polymorphic DNA markers and the reaction products were analyzed for microsatellite instability.

**Results:**

It was found that irrespectively of the applied diet there was no inhibition of mammary carcinogenesis in the rats due to DMBA. Besides, in the groups supplemented with Cu (II) or Cu (II) and resveratrol the tumor formation was clearly accelerated. Unlike the animals that were fed with standard diet, the supplemented rats were characterized by the loss of heterozygosity of microsatellite D3Mgh9 in cancer tumors (by respectively 50 and 40%). When the animals received Cu (II) and resveratrol supplemented diet the occurrence of genomic instability was additionally found in their livers in the case of microsatellite D1Mgh6 (which was stable in the animals without dietary supplementation).

**Conclusions:**

Identification of the underlying mechanisms by which dietary factors affect genomic stability might prove useful in the treatment of mammary cancer as well as in the incorporation of dietary factors into mammary cancer prevention strategies.

## Background

Polyphenolic compounds have been already shown to be chemopreventive against various stages of chemically induced carcinogenesis [1,2]. On the other hand, the procancerogenic activity of copper was found to be related to the formation of reactive oxygen radicals that impair DNA threads and to initiation of angiogenesis of the tumor [3]. Many investigations were performed that confirmed the effectiveness of using copper chelators (e.g. penicillamines, tetrathiomolybdate, zinc anions) as anticancer agents [4,5]. In this research resveratrol was used as the copper chelator because this compound occurs in nature and exhibits strong anti-oxidative properties [2]. The hydroxyl groups of polyphenols, when reacting with free radicals, form more stable complexes. Besides, polyphenols complexate the Cu (II) and Fe (II) ions, and can thus inhibit the activity of enzymes, e.g. of xantine oxidase, and of the processes intensifying the formation of free radicals [1,2,6].

The aim of the present study was to assess the effect of dietary supplementation (copper or copper and resveratrol) on the intensity of carcinogenesis and the frequency of microsatellite instability in a widely used model of mammary carcinogenesis induced in the rat by treatment with 7,12-dimethylbenz[a]anthracene. In the study, we used four different microsatellite polymorphic DNA markers located at 1 (D1Mgh6 127 bp), 3 (D3Mgh7 128 bp; D3Mgh9 125 bp) and 8 (D8Mgh11 146 bp) chromosomes.

## Materials and methods

### Laboratory animals

Female Sprague-Dawley rats were obtained from the Animal Laboratory, Department of General and Experimental Pathology, Medical University of Warsaw. The study was approved by the Ethics Committee, Medical University of Warsaw. After the adaptation period, the animals were divided into two experimental groups. In group 1 (DMBA+, study group n = 30), the rats were treated twice with 80 mg/kg body weight DMBA (7,12-dimethyl-1,2-benz[a]anthracene; Sigma-Aldrich, St. Louis, MO, USA) in rapeseed oil (via gavage) to induce mammary cancer (adenocarcinoma); the first treatment was given at 50 days, followed by the same dose (a repeated dose of 80 mg/kg body weight) at 80 days of rat age, and in group 2 (DMBA-, control group n = 21), the rats were accommodated under the same conditions as those in Group 1; fed the same diet, but without DMBA treatment. Tumors were histopathologically evaluated to confirm their malignancy and to prove that they were *adenocarcinomas *(II and III degree). Spontaneous cancers were not found in the non-DMBA groups.

Rats were housed in an environmentally-controlled room maintained at 22°C with a 12-h light-dark cycle. All animals had free access to water and food (standard diet: Labofeed H, Żurawia 19, 89-240 Kcynia, Poland). Animals from both groups were also supplemented with: copper 1.3 mg/ml (42.6 mg Cu(II)/kg diet) or copper 1.3 mg/ml (42.6 mg Cu(II)/kg diet) and resveratrol 0.1 mg/ml (0.2 mg/kg bw). The rats were fed extra supplements suspended in water (0.4 ml daily via gavage), from 40 days until 20 weeks of age (sacrifice time by decapitation). The resveratrol dose level was selected based on human average daily consumption (extrapolating on the rats' body weight) [7]. The dose of Cu (II) was established based on the values used in the Labofeed H diet (standard diet) (21.3 mg Cu(II)/kg diet) [8]. The copper content in the standard diet was determined following wet microwave sample mineralization using atomic absorption spectrophotometry (AAS). The intralaboratory quality control of determination was based on the following certified Reference Material: NCS ZC 71001 Beef Liver - Cu - 118%; NIST 1577b Bovine liver - Cu - 116%. Limit of detection for Cu was 0.1309 mg/l. The average (mg/kg) of copper content in the standard diet was made of seven determinations: 20.6 ± 6.02.

The animals were examined by palpation during the study to characterize the time course of tumor development. The rats were sacrificed by decapitation at 20 weeks of age, and mammary tumors and livers were collected. The biomaterial samples were stored at a temperature of - 70°C until the test time.

DNA was extracted from 32 rat mammary cancers (randomly chosen for the investigations) and normal tisues (n = 21), amplified by PCR, and analyzed for LOH (loss of heterozygosity) using 10 microsatellite DNA markers (D1Mgh2 150 bp, D1Mgh6 127 bp, D3Mit3 155 bp, D3Mgh5 131 bp, D3Mgh7 128 bp, D3Mgh9 125 bp, D5Mgh5 131 bp, D7Mgh1 126 bp, D7Mgh11 84 bp, D8Mgh11 146 bp). These markers were chosen because they show a high rate of alterations in mammary carcinoma [9]. PCR was performed according to Dahiya et al. [9] modified version of a previously described procedure. Briefly, 1 μl of DNA was amplified by PCR in 25.5 μl of solution containing 2.5 mM each dNTPs; 5 μl upstream and downstream primers, 1 μl of RUN polymerase (1 U/μl) in PCR buffer containing 1.5 mM MgCl_2_. The PCR reaction was started at 94°C for 3 min, followed by 40 cycles of denaturing (94°C for 30 s), annealing (56°C for 30 s) and chain extension (72°C for 30 s), followed by 5 min at 72°C. The primers used to amplify simple repeated sequences were synthesized according to previously published sequences [9]. The PCR product was electrophoresed on a 12% polyacrylamide gel.

## Results and discussion

The copper dose applied (exceeding twice the dose in the feedstuff) did not result in any loss of appetite or loss of body mass of the rats. Moreover, in the group supplemented with Cu(II) and resveratrol the body mass of the rats considerably increased (Table 1) (data are given as means ± SD. The data were analyzed using Student's t-test; differences at p ≤ 0.05 were considered statistically significant). Irrespectively of the diet applied the effectiveness of cancer induction by DMBA was 100%. There were both single and multiple tumors - maximally 6 per rat. In the groups of rats supplemented with Cu(II) or Cu(II) and resveratrol the first tumors felt by palpation appeared a week earlier than in the group fed a standard diet (which corresponds to a period of about 6 months in human life) [10] (Table 1). The increased Cu(II) concentration was found in different types of tumors [11,12]. The amount of resveratrol used in our investigations had no protective effect (probably because of a high additional supply of copper ions) on the process of tumor formation.

**Table 1:** Tumor induction in 7,12-dimethylbenz[a]anthracene treated groups in relation to diet.

Diet	Tumor incidence (%)^1^	Number of tumors in one rat	Maximum tumor weight in one rat in group (g)	First week at onset	Body weight ± SD
1. Standard diet	9/9 (100)	1-5	12	15	206 ± 8*
2. Copper 1.3 mg/ml (42.6 mg Cu/kg diet)	9/9 (100)	1-5	7	14	231 ± 22
3. Copper 1.3 mg/ml (42.6 mg Cu/kg diet) and resveratrol 0.1 mg/ml (0.2 mg/kg bw)	12/12 (100)	2-6	11	14	229 ± 13*

The data refer to tumors evaluated at 20 weeks of the animals' age (decapitation time).

^1 ^Tumor incidence is expressed as the number of tumor-bearing rats over the total number of rats in the group.

* - statistical significance p < 0.05

As a result of investigations performed in rats that received only a standard diet the microsatellite instability occurred in the livers in the case of 2 out of 10 microsatellites (D3Mgh7 128 bp and D3Mgh9 125 bp) (Table 2). In the case of two liver samples the loss of heterozygosity occurred in 2 loci. The loss of heterozygosity in the livers occurred in 2 out of 4 microsatellites on chromosome 3. For all of the analyzed markers no microsatellite changes were found in the case of cancer tumors. The occurrence of a larger number of instabilities in the control material as compared with the cancer tumors in different types of cancers, including mammary cancer, was also found by other authors [13]. Dahiya et al. [9] examined 27 different microsatellite markers occurring on chromosomes 1,3,5,7,8 DNA isolated from the rats with mammary cancer induced by methylnitrosourea (50 mg/kg body weight). They showed that in 30% cases (9/30) of the examined samples the microsatellite instability occurred in at least 1 locus: six cases of MI (microsatellite instability) were found for loci D5Mit11, D5Mgh3, five for loci D1Mit14, D1Mgh6, D5Mgh5, D8Mgh10, four for loci D1Mgh2, D3Mgh7, two for loci D7Mit11. Toyota et al. [14] investigated the microsatellite instability of 62 DNA markers of tumors in rats that were treated with 2-amino-1-methyl-6-phenylimidazo[4,5-b]pyridine (PhIP) or 7,12-dimethylbenzo[a]anthracene in order to induce mammary cancer (adenocarcinoma). The changes in length of the analyzed sequences occurred in 60% (9/15) of the samples originating from the animals treated with PhIP and in 17% (2/12) of the samples originating from the animals treated with DMBA, including mutations in 4 loci: D3Mgh9, D9Mit3, D20Mgh1, TNF. The last one was found only in one analyzed sample, originating from the rat treated with PhIP. In the case of the animals treated with DMBA the instabilities occurred in loci: D15Mgh4 and D18Mgh3, whereas in the case of rats supplemented with PhIP MI was found in loci: D3Mgh9, D9Mit3, D20Mgh1, TNF, D6Mgh3, D20Mit1, PND, MT1PA, DXmgh1, TNF, D2N91, D16Mgh3, RBP-2, D6Mgh7, D2Mit2, TAT, REN, D14Mgh2. From the investigations performed it results that microsatellite instability in mammary cancers induced with DMBA occurs rather rarely in comparison with the case when other chemical carcinogens were applied. This reduces the application of the model to nutrigenomic studies only.

**Table 2:** Summary of MSI analysis for DMBA-induced mammary carcinoma and liver of rat fed only the standard diet (without supplementation)

Marker	Tumor (n = 14) Number of rat/Tumor determination														Liver (n = 6) Number of rat					
	9/3	9/4	9/2	8/1	6/3	4/3	7/1	1/3	9/1	5/4	3/2	4/2	6/2	7/2	1	2	3	4	5	6
D1Mgh2 150 bp	**+**	**+**	**+**	**+**	**+**	**+**	**+**	**+**	**+**	**+**	**+**	**+**	**+**	**+**	**+**	**+**	**+**	**+**	**+**	**+**
D1Mgh6 127 bp	**+**	**+**	**+**	**+**	**+**	**+**	**+**	**+**	**+**	**+**	**+**	**+**	**+**	**+**	**+**	**+**	**+**	**+**	**+**	**+**
D3Mit3 155 bp	**+**	**+**	**+**	**+**	**+**	**+**	**+**	**+**	**+**	**+**	**+**	**+**	**+**	**+**	**+**	**+**	**+**	**+**	**+**	**+**
D3Mgh5 131 bp	**+**	**+**	**+**	**+**	**+**	**+**	**+**	**+**	**+**	**+**	**+**	**+**	**+**	**+**	**+**	**+**	**+**	**+**	**+**	**+**
D3Mgh7 128 bp	**+**	**+**	**+**	**+**	**+**	**+**	**+**	**+**	**+**	**+**	**+**	**+**	**+**	**+**	L	L	L	L	L	L
D3Mgh9 125 bp	**+**	**+**	**+**	**+**	**+**	**+**	**+**	**+**	**+**	**+**	**+**	**+**	**+**	**+**	**+**	+	L	L	+	+
D5Mgh5 131 bp	**+**	**+**	**+**	**+**	**+**	**+**	**+**	**+**	**+**	**+**	**+**	**+**	**+**	**+**	**+**	**+**	**+**	**+**	**+**	**+**
D7Mgh1 126 bp	**+**	**+**	**+**	**+**	**+**	**+**	**+**	**+**	**+**	**+**	**+**	**+**	**+**	**+**	**+**	**+**	**+**	**+**	**+**	**+**
D7Mit11 84 bp	**+**	**+**	**+**	**+**	**+**	**+**	**+**	**+**	**+**	**+**	**+**	**+**	**+**	**+**	**+**	**+**	**+**	**+**	**+**	**+**
D8Mgh11 146 bp	**+**	**+**	**+**	**+**	**+**	**+**	**+**	**+**	**+**	**+**	**+**	**+**	**+**	**+**	**+**	**+**	**+**	**+**	**+**	**+**

L - loss of heterozygosity

+ - genomic stability

On the basis of results obtained in the case of animals fed a standard diet, for further investigations of the effect of supplementation on microsatellite instability 4 microsatellites were chosen, including two in which some changes in genetic material were found (D3Mgh7 and D3Mgh9) as well as 2 microsatellites in which no instability occurred (D1Mgh6 and D8Mgh11) (randomly chosen for the investigations). It was interesting to note that the animals that were supplemented with Cu (II) or Cu (II) with resveratrol were characterized by the loss of heterozygosity of microsatellite D3Mgh9 in cancer tumors (depending on the kind of supplementation by, respectively, 50% and 40%) (Tables 3 and 4). In the case when the animals were fed a diet supplemented with Cu (II) and resveratrol, additionally in the livers of rats the instability of microsatellite D1Mgh6 (2 of 8 samples (25%)) was observed. Generally, it was found that the mobilization of endogenous copper, that is bound in the nucleus by a substance like resveratrol, may result in the damage and DNA breakage of cancer cells in humans. This was also confirmed by other investigations [11,15]. In those investigations it is important to choose the appropriately high dose of resveratrol and the correct time of supplementation [2,11,16]. According to some reports, the supply of resveratrol (which shows some similarity to sex steroid hormones) before pubescence results in endocrine disturbances causing irregular cycles with prolonged estrus phase, thus increasing the multitude of mammary tumors in rats. It should be strongly emphasized that resveratrol exhibits anticancerogenous activity on animal models when it is administered to adult animals [17]. There are a number of papers, in particular those performed on cell lines, concerned with the effect of polyphenols, including resveratrol, in the presence of Cu (II) on the genetic material. Resveratrol when administered at concentrations of 25-200 μM increases DNA breakages induced by H_2_O_2_/Cu(II). The authors of many studies point out that resveratrol in the presence of Cu (II) ions increases DNA damage. The cancer cells accumulate large amounts of copper, including that bound in the nucleus. As a result the obtained resveratrol/Cu (II) complex after joining with DNA may induce its breakage and lead to apoptosis [18]. However, in the applied research model, the addition of resveratrol in a dose of 0.2 mg/kg body weight, did not effectively inhibit the rate of tumor formation, probably due to supplementation with copper ions, or to a more intensive process of cancer development as a result of a high dose of DMBA applied.

**Table 3:** Summary of MSI analysis for DMBA-induced mammary carcinoma and liver of rats fed the diet supplemented with copper 1.3 mg/ml (42.6 mg Cu/kg diet)

Marker	Tumors (n = 8) Number of rat/Tumor determination								Livers (n = 7) Number of rat						
	3/3	2/1	6/2	1/1	1/3	7/1	6/3	8/2	1	2	3	4	5	6	7
D1Mgh6 127 bp	+	+	+	+	+	+	+	+	+	+	+	+	+	+	+
D3Mgh7 128 bp	+	+	+	+	+	+	+	+	+	+	+	+	+	+	+
D3Mgh9 125 bp	L	+	+	L	L	+	+	L	+	+	+	+	+	+	+
D8Mgh11 146 bp	+	+	+	+	+	+	+	+	+	+	+	+	+	+	+

L - loss of heterozygosity

+ - genomic stability

**Table 4:** Summary of MSI analysis for DMBA-induced mammary carcinoma and liver of rats fed the diet supplemented with copper 1.3 mg/ml (42.6 mg Cu/kg diet) and resveratrol 0.1 mg/ml (0.2 mg/kg bw)

Marker	Tumor (n = 10) Number of rat/Tumor determination										Liver (n = 8) Number of rat							
	2/2	11/1	4/2	5/5	7/3	8/1	8/4	9/1	9/2	10/2	1	2	3	4	5	6	7	8
D1Mgh6 127 bp	+	+	+	+	+	+	+	+	+	+	+	+	L	+	+	L	+	+
D3Mgh7 128 bp	+	+	+	+	+	+	+	+	+	+	+	+	+	+	+	+	+	+
D3Mgh9 128 bp	L	L	+	L	L	+	+	+	+	+	+	+	L	+	+	+	+	L
D8Mgh11 146 bp	+	+	+	+	+	+	+	+	+	+	+	+	+	+	+	+	+	+

L - loss of heterozygosity

+ - genomic stability

The dependence of the effect of mineral components on the occurrence of microsatellite instability was also shown by other authors. Numerous epidemiologic studies have established that chromium compounds are potent carcinogens. El-Ghor et al. [19] investigated, based on the rat model, the effect of oral acute and subchronic doses of cadmium chloride on microsatellite instability at D6Mit3, D9Mit2, D15Mgh1 loci. In the acute study, no MSI was observed in all three tested loci. In subchronic study, the MSI was observed in three studied loci and was in the form of deletion of 2-3 bp or addition of 3-6 bp. Sato et al. [20] compared amplified DNA samples from the second tumor specimen with normal constitutional DNA (normal nasal mucosa and blood lymphocytes) from a 56-year-old patient who had worked at a chromate factory for 13 years. They did not detect microsatellite instability at any of the four loci tested. Loss of heterozygosity at 2p, 3p, 17p and 18q loci was not seen either. Wei et al. [21] analyzed eighteen microsatellite loci interspersed throughout the rat genome in 16 dimethylarsenic acid (DMA)-induced urinary bladder carcinomas, but no alterations were detected either. DMA is a major metabolite of arsenic in most mammals including humans. Slebos et al. [22] demonstrated that exposure of the human fibrosarcoma cell line HT1080 to cadmium led to statistically significant increases in microsatellite mutations. Zienolddiny et al. [23] suggested that Ni (II) may induce microsatellite mutations by creating a hypermutable state that may be of importance in tumor progression. In the literature there are no data concerning the effect of some mineral components that are indispensable for proper functioning of human organism (such as zinc, calcium, copper, magnesium or iron as well as polyphenolic compounds) on the occurrence of microsatellite instability. And these data would be very important because identification of the underlying mechanisms by which dietary factors affect genomic stability might prove useful in the treatment of mammary cancer as well as in the incorporation of dietary factors into mammary cancer prevention strategies.

## Conclusions

Summing up the results presented in this paper the following conclusions can be drawn:

The amount of resveratrol used in our investigations had no protective effect on the process of tumor formation.

The frequency of occurrence of microsatellite instability depends on the kind of supplementation applied.

The mobilization of endogenous copper, that is bound in the nucleus by a substance like resveratrol, may result in the damage and DNA breakage of cancer cells in humans.

## List of abbreviations

AAS: atomic absorption spectrophotometry; Cu: copper; DMA: dimethylarsenic acid; DMBA: 7,12-dimethylbenz[a]anthracene; Fe: iron, LOH loss of heterozygosity; Ni: nickel; PCR: polymerase chain reaction; PhIP: 2-amino-1-methyl-6-phenylimidazo[4,5-b]pyridine.

## Competing interests

The authors declare that they have no competing interests.

## Authors' contributions

BB planned, designed and carried out the experiment and performed the statistical analysis. DS planned, designed and carried out the experiment and performed the statistical analysis AT coordinated the study. All authors read and approved the final manuscript.
